# Insulinoma Unmasked By Tirzepatide: A Rare Case of Postprandial Hypoglycemia In a Nondiabetic Patient

**DOI:** 10.1210/jcemcr/luaf290

**Published:** 2025-12-04

**Authors:** Michael Polisky, Dina Kamel, Jeong-hee Ku

**Affiliations:** Division of Endocrinology, Diabetes, and Metabolism, Department of Medicine, UCLA David Geffen School of Medicine, Los Angeles, CA 90095, USA; Division of Endocrinology, Diabetes, and Metabolism, Department of Medicine, UCLA David Geffen School of Medicine, Los Angeles, CA 90095, USA; Division of Endocrinology, Diabetes, and Metabolism, Department of Medicine, UCLA David Geffen School of Medicine, Los Angeles, CA 90095, USA

**Keywords:** insulinoma, postprandial hypoglycemia, tirzepatide, neuroendocrine tumor, GLP-1 receptor agonist, GIP receptor agonist

## Abstract

A 63-year-old woman with obesity presented with severe postprandial hypoglycemia that worsened after starting tirzepatide for weight loss. Further evaluation led to the diagnosis of insulinoma. This case suggests that the use of tirzepatide can provoke severe hypoglycemia episodes in patients with insulinoma and highlights the importance of including insulinoma as a differential diagnosis for hypoglycemia in patients taking incretin-based therapy.

## Introduction

Although rare, insulinoma is one of the diagnoses that should be considered in the evaluation of hypoglycemia in patients without diabetes. Other possible causes of hypoglycemia include critical illness, postbariatric hypoglycemia, alcohol abuse, adrenal insufficiency, and inappropriate use of insulin secretagogues. Typically, insulinoma presents as fasting hypoglycemia or unpredictable hypoglycemia; however, exclusive presentation of postprandial hypoglycemia has been described in rare cases [1].

Tirzepatide is a dual incretin receptor agonist that activates both glucagon-like peptide-1 (GLP-1) and glucose-dependent insulinotropic peptide (GIP) receptors. Along with semaglutide and liraglutide, it belongs to a class of incretin mimetics initially developed for managing hyperglycemia in type 2 diabetes and later approved for the treatment of obesity. Although these agents are associated with a lower risk of hypoglycemia compared to other diabetes medications such as sulfonylureas or insulin [2], hypoglycemia remains a reported side effect—even in clinical trials involving patients with obesity and no underlying diabetes [3, 4].

To date, only a limited number of cases describing the diagnosis of insulinoma after the use of GLP-1 receptor agonists (GLP-1 RAs) have been reported in the literature [5-7]. We report a case of insulinoma diagnosed in a patient without diabetes who was taking tirzepatide for weight loss.

## Case Presentation

A 63-year-old woman with a past medical history significant for obesity presented for evaluation of recurrent hypoglycemia. She had no exposure to insulin secretagogues and no prior gastrointestinal surgical history. The patient's symptoms began 6 months before presentation and consisted of word-finding difficulty, perioral tingling, and dizziness, which typically occurred during the afternoon. Initially, she attributed these episodes to skipping meals, as her symptoms improved after eating. However, she later noted that the symptoms occurred in the afternoon even after consuming lunch. Notably, these episodes did not present overnight or in the early morning. Four months before presentation, she started tirzepatide for weight loss, escalating from 2.5 to 5 mg weekly. After resuming the 5-mg dose following a brief interruption due to nausea and vomiting, she became confused and unresponsive, with a blood glucose of 36 mg/dL (SI: 2 mmol/L) (reference range, 65-99 mg/dL [SI: 3.6-5.4 mmol/L]) in the emergency department. C-peptide measured the next morning was 2.16 ng/mL (SI: 0.71 nmol/L) (reference range, 1.1-4.3 ng/mL [SI: 0.36-1.4 mmol/L]) with a concurrent glucose of 130 mg/dL (SI: 7.71 mmol/L). Abdominal computed tomography (CT) without contrast was unremarkable. Tirzepatide was discontinued, and she was discharged with a glucose meter and referred to endocrinology. After discharge, she continued to experience near-daily afternoon hypoglycemia, with glucose readings as low as 43 mg/dL (SI: 2.39 mmol/L).

## Diagnostic Assessment

Other than obesity with body mass index of 36.2, the patient’s physical examination was unremarkable.

Given her history of near daily occurrence of hypoglycemia in the afternoons, she was directed to undergo fasting laboratory testing in the afternoon with the hope of capturing a hypoglycemia episode and avoid a 72-hour fast. She was educated on signs and symptoms of hypoglycemia and its treatment prior. Initial laboratory results demonstrated clear endogenous hyperinsulinemic hypoglycemia, with a fasting glucose 45 mg/dL (SI: 2.5 mmol/L), concomitantly elevated insulin and C-peptide levels, and a negative sulfonylurea screen (see Table 1). Therefore a 72-hour fast was deemed unnecessary and the patient proceeded with a localization study.

**Table 1.** Laboratory evaluation

Laboratory test	Results	Reference range
Glucose	45 mg/dL (2.5 mmol/L)	65-99 mg/dL (3.61-5.49 mmol/L)
Insulin	15 μU/mL (104.2 pmol/L)	3-25 μU/mL (20.8-173.6 pmol/L)
C-peptide	2.9 ng/mL (0.957 nmol/L)	1.1-4.3 ng/mL (0.36-1.42 nmol/L)
β-Hydroxybutyrate	1.2 mg/dL (0.1153 mmol/L)	<3 mg/dL (<0.29 mmol/L)
Proinsulin	2.4 pmol/L (2.4 pmol/L)	<7.2 pmol/L (<7.2 pmol/L)
Cortisol	15 μg/dL (414 nmol/L)	8-25 μg/dL (220-690 nmol/L)
IGF-1	115 ng/mL (15 nmol/L)	41-279 ng/mL (5.4-36.5 nmol/L)
IGF-2	428 ng/mL (55.9 nmol/L)	180-580 ng/mL (23.4-75.4 nmol/L)
Sulfonylurea metabolites	Negative	Negative
Insulin antibody	<0.4 U/mL (<0.4 kU/L)	0-0.4 U/mL (0-0.4 kU/L)

Values in parentheses are International System of Units (SI).

Abbreviations: IGF-1, insulin-like growth factor 1; IGF-2, insulin-like growth factor 2.

**Figure 1.**
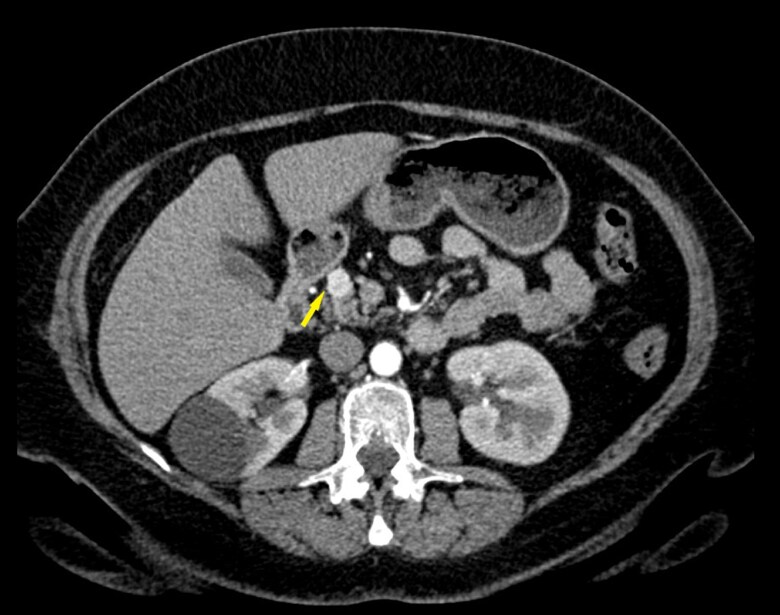
Contrast-enhanced computed tomography of the abdomen demonstrating a 1.4-cm mass in the pancreatic neck (arrow).

For the imaging study, patient had a CT scan of abdomen with intravenous contrast, which showed a 1.4 × 1.1 × 1.1-cm mass arising from the pancreatic neck as shown in Fig. 1. The patient was referred to endocrine surgery for surgical consultation.

## Treatment

The patient was admitted to the hospital and underwent an elective robotic-assisted pancreatic tumor enucleation with intraoperative ultrasound. A 1.8 × 1.4-cm tumor was identified and enucleated. The postoperative course was uncomplicated. During the first 24 hours, the patient was noted to be mildly hyperglycemic, with glucose values ranging as high as 205 mg/dL (SI: 11.38 mmol/L). By postoperative day 2, her glucose levels had stabilized within the 99 to 113 mg/dL (SI: 5.49-6.27 mmol/L) range, and she was discharged home. Pathology confirmed a 1.5-cm grade 1 neuroendocrine tumor (NET).

## Outcome and Follow-up

At her 11-week postoperative follow-up, the patient reported feeling better immediately after surgery, with resolution of hunger and improvement of cravings. She did not experience any further episodes of hypoglycemia.

## Discussion

This case is notable for two key features: (1) insulinoma presenting primarily with postprandial hypoglycemia and (2) symptom exacerbation following initiation of tirzepatide, a dual GIP/GLP-1 RA. Exclusive postprandial hypoglycemia is a rare presentation of insulinoma, reported in only 6% of 237 patients in a large retrospective analysis [1], while 73% presented solely with fasting hypoglycemia, and 21% reported both fasting and postprandial hypoglycemia. A proposed mechanism for this type of presentation is exaggerated insulin secretion in response to postprandial GLP-1 release [8].

GLP-1 and GIP are incretin hormones that regulate insulin secretion in a glucose-dependent manner [9]. While incretin-based therapies, such as tirzepatide, are generally associated with a low risk of hypoglycemia, they can stimulate insulin secretion in susceptible individuals. GLP-1 and GIP receptors are expressed not only in normal pancreatic islets but also in insulinomas, with studies showing that benign insulinomas frequently overexpress GLP-1 receptors [10], and both benign and malignant insulinomas express GIP receptors [11]. The presence of these receptors has been leveraged for tumor localization [12, 13] and may also explain why incretin mimetics can provoke or unmask hypoglycemia in patients with insulinoma.

Similar cases in the literature include a patient with type 2 diabetes who developed severe hypoglycemia after starting liraglutide, later found to have a GLP-1 receptor–positive insulinoma [5]. GLP-1 receptor activation was a suspected mechanism for the rapid onset of hypoglycemia in that case. In our patient, mild symptoms predated tirzepatide initiation, but hypoglycemia became more severe and culminated in an emergency presentation shortly after resuming the drug, suggesting a possible amplifying effect. If insulinomas overexpress GIP and GLP receptors, use of tirzepatide could intensify postprandial insulin production and provoke severe postprandial hypoglycemia.

Given the overexpression of GLP-1 and GIP receptors in pancreatic NETs, there is a concern for a possible causal relationship between incretins and NETs. There has been an in vitro study showing semaglutide promoting NET cell proliferation [14]. However, large observational studies including patients with type 2 diabetes and obesity have suggested improved survival outcomes in patients with preexisting NETs using GLP-1 or GIP/GLP-1 RAs [15]. Nevertheless, additional studies are needed to better understand the long-term effects of these agents on tumor biology.

Hypoglycemia is a recognized adverse effect in clinical trials involving obese patients without diabetes, with reported incidences of 2.6% for semaglutide [3] and 1.4% for tirzepatide [4]. It remains unclear whether these events are due to decreased oral intake from appetite suppression or gastrointestinal side effects, or whether hypoglycemia resolves after discontinuation of the medication. Although only a few cases of insulinoma diagnosed following the use of GLP-1 or GIP/GLP-1 RAs have been reported [5-7], the potential for these agents to unmask or exacerbate insulinoma-related hypoglycemia warrants further investigation. Given the expanding use of incretin-based therapies for obesity, clinicians should maintain a high index of suspicion for insulinoma in patients presenting with severe or persistent hypoglycemia.

## Learning Points

- Incretin-based therapy including GLP-1 RAs as well as GIP and GLP-1 dual-RAs may exacerbate the severity of hypoglycemic episodes from an existing insulinoma, as insulinoma commonly overexpresses GIP and GLP-1 receptors.
- It is imperative for clinicians to evaluate hypoglycemia events further if patients develop them after starting GIP/GLP-1 RAs.
- When a patient who develops hypoglycemia with GIP/GLP-1 RA therapy continues to experience hypoglycemia after discontinuation of the drug, insulinoma should be ruled out.

## Contributors

All authors made individual contributions to authorship. M.P. was involved in the diagnosis and management of the patient and developed the clinical description of the case. D.K. and J.K. carried out the literature review, interpreted the clinical findings, and revised the manuscript for important intellectual content. All authors reviewed and approved the final manuscript.

## Data Availability

Data sharing not applicable to this article as no datasets were generated or analyzed during the current study

## Abbreviations

CT: computed tomography
GIP: glucose-dependent insulinotropic peptide
GLP-1: glucagon-like peptide-1
GLP-1 RAs: GLP-1 receptor agonists
IGF-1: insulin-like growth factor 1
IGF-2: insulin-like growth factor 2
NET: neuroendocrine tumor
